# Round the Hospitals

**Published:** 1919-10-11

**Authors:** 


### ROUND THE HOSPITALS.

We are asked to announce that the Council of the College of Nursing is prepared to make grants to members to enable them to qualify as midwives. Any members wishing to avail themselves of this opportunity should write at once for application forms to the Secretary, the College of Nursing, Ltd., 7 Henrietta Street, Cavendish Square, W. 1. We have no doubt that there are many members of the College who will eagerly seize this opportunity to add to their qualifications.

**ROUND THE HOSPITALS**—*(continued)*.

The influence of the College of Nursing continues to extend, and it is now proposing the election of a "Supervisor of Nursing Studies," relative to students qualifying for the posts of sister-tutors. The eight successful candidates of this year of the last examination, whose names we gave in our issue of July 26, are now in residence at the King's College for Women, Campden Hill, and Miss Bannon, one of the successful students of last year, is to continue her studies for a second year, specialising upon subjects in connection with public health. At the end of the second year she will, if successful, be appointed on the staff, and, under the direction of the Dean, Dr. Janet Lane-Claypon, who is so well known for her sympathetic interest and help in all matters concerning the College, and in forwarding educational advantages for nurses, will assist and advise student nurses in their several studies. The expenses incurred by Miss Bannon's second year are being borne by the different Local Centres of the College, thus enabling members to assist in furthering its educational developments and to take an active and lively interest in whatever schemes the College may undertake for their mutual benefit and better advantage. Necessarily some time must elapse before a degree of nursing can be established, but a great step has been made in the right direction in at once qualifying a trained nurse to undertake the supervision of studies of the nurse-students at King's College.

---

Nursing sisters in all parts of the world owe a great debt of gratitude to Sir Edward Tyas Cook, who died at his beautiful riverside home at Southstoke on September 30, after a short illness. To his competent hands was entrusted the honourable task of writing Florence Nightingale's "Life," and certainly it could have fallen to no one better qualified. Few readers guess at the mass of documents and State papers which it was necessary to master before this chapter of history could be written. The story of the founding of modern nursing lay hidden in tumultuous Parliamentary controversies. On the one hand was the danger of blurring the figure of Florence Nightingale under the shadow of a peculiarly bitter personal struggle; on the other that of watering down her achievements to the level of ordinary philanthropic impulse. Sir Edward Cook chose his material with rare skill, and his work forms the corner-stone of the history of nursing in the nineteenth century. His portrait of Florence Nightingale, though differing in many important respects from the legendary figure, bears the impress of truth, and sets this great woman before us in the exercise of her full powers. It is a revelation of the inspiration and force of her nursing ideals and the secret of her success.

---

Dr. Addison gave a very striking address at the opening of the session on October 1 at the Royal Free Hospital. In particular he laid great stress on the miserable remuneration given to nurses, "who are paid less than cooks and scullery-maids." This declaration on the part of the Minister of Health ought to put good heart into nurses. It is clear that he recognises the extent to which the success of his Department depends on a well-paid, well-educated, and efficient nursing service. The wastage due to ill-health in every branch of national work (270,000 workers annually on the sick list) can only be reduced by the ceaseless vigilance of nurses working under the medical officers of health, and it is so obvious as to be generally overlooked that nurses who are overworked and ill-paid, who are drudges instead of active coadjutors, can only add to this deplorable state of affairs, instead of helping to remedy it.

---

By common consent the voluntary workers in the days of trial during the strike set themselves first of all to mitigate its inconveniences to the hospitals. So far no complaint has reached us of shortage of milk or other necessaries for the sick, though at Sheffield some difficulties occurred. But minor discomforts have been numerous. Many nurses were due to return from their holidays when the news first broke upon the public that the railways were closed. But it is not easy to daunt the woman who knows that her ward is awaiting her, and by hook or by crook, by motor, carriage, or cart, by bicycle, or even on foot, the stragglers made their way back to their patients. The effect of being thrown on their own resources is curiously exhilarating to some natures, and while there was a good deal of natural impatience among those marooned far from home, there was nothing but smiles on the faces of those who got under way.

---

An infirmary matron writes to the *Morning Post* deploring the very serious shortage of candidates for Poor-Law infirmaries. We are entirely in accord with her eulogies of the training provided in the best infirmaries. She declares that the nature of the work, the treatment of acute and chronic cases of all kinds, the extensive experience, the variety of patients, drawn from many social classes, all combine to make the training particularly well fitted for women wishing to take up district, private, or colonial nursing or service in public health and social betterment undertakings. She puts her finger on the weak point in the existing nursing organisation when she urges that "there must be many young women with a taste and capacity for nursing who would come forward *if they knew how badly they were needed.*" It is the deplorable ineptitude of our methods as regards candidates which are responsible for the present shortage. For many years past the policy has been discouragement owing to the overwhelming number of applicants Nothing will serve now but systematic and intelligent recruiting at a Central Nursing Bureau in London and at similar offices in all large provincial centres.

---

Many Metropolitan Poor-Law institutions have reduced the age limit for probationers, owing to the

**ROUND THE HOSPITALS—(continued).**

present shortage of hands, and the Southwark Guardians Infirmary Committee have decided to follow suit and reduce the age for the present from twenty-one to nineteen. The voluntary hospitals are also far less rigid than they used to be on this point, and no likely candidate is now rejected because her years do not exactly tally with the rules. Many experienced matrons believe that this is all for the good, and that the modern young woman is as well prepared for her duties at twenty as her predecessors were at twenty-three. From the nurse's point of view the addition of another two or three years to her earning period is a boon indeed.

At Hastings the fine new Poor-law buildings, in which the sick wounded were housed during the war, have been evacuated, and the result of the military occupation has been to effect many improvements, including the installation of a good operating-theatre. It is hoped to get the sick poor out of the old workhouse buildings and into their own quarters by the beginning of the New Year, and we learn with much pleasure that it is in contemplation to engage a resident medical officer and inaugurate a training-school for nurses. There will be nearly 300 beds when the building is re-fitted.

Among volunteer nurses without much preparation for their duties, Miss Valentine, whose adventures have lately been the theme of the lay press appears to have been remarkable both in the chances of being useful which came in her way and also let it be said in the admirable spirit in which they were encountered. Miss Valentine's equipment for her tasks is described as "three years in an English college and some training in English nursing." She joined her father and sister in Petrograd at the moment when the revolution was beginning and subsequently made her way to the British Army headquarters at Archangel. Here, "the only woman nurse on the Dvina front," she became matron (no less) of a hospital and hospital barge at Berniski, where courage and resource carried her through. One of her memorable experiences lay in assisting with four Red Cross men and a doctor to bring in fifty wounded men by navigating the barge through the Bolshevik lines before daylight. No one will grudge her the Military Medal won by this exercise of nerve and resolution. She has also been awarded the Order of the Star of Russia.

The general desire of the fortunate people "mentioned in despatches" to have some official record of the fact has been met by the issue of certificates which will, we trust, be available for members of the nursing services as well as for officers, non-commissioned officers, and men. The certificate, $8\frac{1}{2}$ by $7\frac{1}{2}$ inches, is on white cardboard, well adapted for framing. It is headed with the Royal Arms, and after defining the status of the recipient and the date when mentioned, continues: "I have it in command from the King to record His Majesty's high appreciation of the services rendered." It is signed Winston S. Churchill, Secretary of State for War. Such a certificate, ranking as one of the most precious possessions of the recipient, at once removes the tinge of disappointment experienced at merely reading one's name in a closely printed column of very small print, after the recognition of heroic exertions by the Commander-in-Chief, and restores the due sense of distinction at the honour conferred.

One of the best known of children's hospitals under the M.A.B. is shortly to undergo some extensive changes, and in addition to being enlarged will also change its name. Attention is drawn to the appointment columns, where some very interesting announcements are made concerning the new staff to be attached to the hospital hitherto known as the East Cliff House, Cliftonville, Margate. In the selection of the new staff a careful survey has been made, and many of our best and well-known training-schools are represented. The acquisition of this high order of talent should ensure the future of the hospital to rank as a first-class institution for the treatment of children suffering from non-pulmonary tuberculosis. The hospital has 270 beds, and is to be equipped throughout on the most modern lines.

From Melbourne comes the news that the State War Council has decided to contribute £50 a year for two years towards the support of a bush nurse at Manangatan in the Mallee Country. The whole of the country between Manangatan and Bryde's Tank, the terminus of the authorised extension of the railway, will probably be occupied by returned soldiers, for whom the surrounding parishes have been exclusively reserved, and a considerable proportion of them are married men with families. The defence authorities have lately compiled a return showing that 2,379 nurses embarked for war service from Australia, while other 588 were enrolled for home service. Of the 21 nurses who died as the result of war service, 12 were Victorians. Matron J. McHardie White, O.B.E., R.R.C., returned recently from Salonica, where she had been matron-in-chief for nearly two years. During the Gallipoli campaign she did fine work under eminent surgeons, and contributed largely to the admirable organisation of the Australian Nursing contingent.

The Ladies' Needlework Guild of the Cossham Memorial Hospital, Kingswood, was formed five years ago, and did a great deal of work at high pressure during the war, when the wards were filled with sailors and soldiers. It is not ceasing its activities now that the hospital has returned to its normal functions among the sick poor, and an interesting exhibition of the work done during the present year was recently opened by the Lord Mayor and Lady Mayoress of Bristol. It is most satisfactory that the work started in the great national crisis should be carried on into years of peace, and become a permanent feature of the institution.

**ROUND THE HOSPITALS**—*(continued).*

The President, Committee, and Matron (Miss Bagnall) of the Royal Southern Hospital, Liverpool, made a joint effort last week on behalf of the Nation's Fund for Nurses. A meeting was held at the Midland Adelphi Hotel, Liverpool, at which Mr. Thomas Woodsend, President of the Royal Southern Hospital, presided. Mrs. Martin Harvey spoke on the aims of the Fund, and Mr. Martin Harvey recited. The two main things so far accomplished by this valuable Fund have been the establishment of scholarships for the training of Sister-Tutors at the University of London and the permanent relief of many nurses shattered mentally or physically who have bought health for others at the expense of their own. The promotors of the Fund look to the extension of these good works in the future, to the development of local centres for nurses in all parts of the country, and to the provision ultimately through the operations of the College of Nursing of adequate payment for nurses. Mr. Galsworthy's fine appeal for the Nation's Fund cannot be too widely circulated.

Sir Jesse Boot (Boots, Ltd.) was well inspired when at a critical moment in the strike he appealed to the pilots of the Aero Company at Nottingham to postpone their public flying display at the Trent aerodrome and carry an urgently needed supply of drugs to Grantham and Lincoln instead. People assembled on the grounds gave vigorous demonstrations of their approval when the cases were brought down and packed into the machines. Grantham was reached in 17 minutes, and Lincoln, after a brief delay, another 20 minutes, and the aviator, Lieutenant MacRae, was loudly cheered on his return. The problem of getting supplies forwarded for the sick was thus very cleverly solved. Considerable difficulty has been experienced at Sheffield in getting anæsthetics through to the hospitals, and we understand that in some cases operations have had to be postponed.

Mrs. Creagh, O.B.E., R.R.C., matron of the South African Nursing Contingent which came over in the autumn of 1915, has now returned to Cape Town, and has met with a fitting welcome. She first went to South Africa to nurse the sick and wounded in the Boer War, and remained there, filling more than one important post until she brought her contingent over, first to England and then to France. The South African Base Hospital at Abbeville, of which she was matron, became in course of time a "fractured femur base," and so remained until after incessant bombing it was decided in 1918 to remove such cases to Netley. At the time of its greatest expansion it contained, including tents, 1,700 beds. Finally, just as danger was over, dug-outs with sleeping accommodation came into existence. During the German push at Easter last year the South African Hospital was rapidly evacuated, and the matron remained behind with seven sisters to cope with the inrush of the wounded from Amiens. From then till some hours after the Armistice was signed the work continued at high pressure, and not till last May was Mrs. Creagh set free from the duties she had so ably and whole-heartedly performed. Like all the good matrons we have ever heard of, she represents herself as "particularly fortunate in her staff."

The Monmouthshire Nursing Association has come into a valuable property through the beneficence of Mayor Beynon, Director of the British Red Cross Society for that county. Mayor Beynon has long had the needs of the poorer mothers in his mind, and has purchased Troedrhien Fawr, Newbridge, a large house standing in three acres of ground with some adjoining property, for the purpose of making it into a Maternity Hospital for the county. The premises are to be thoroughly re-fitted and provided with all modern requisites, and an operating-theatre is to be added. The situation is admirable, commanding fine views, and when completed the Home will offer splendid accommodation. The total cost is estimated at nearly £10,000. The Monmouthshire Nursing Association recently came into £3,000 from the B.R.C.S., and its influence and powers for good will be greatly extended by this new gift, which is being presented through the County Council.

Many V.A.D. grumbles have made themselves heard at various times. But the male V.A.D. workers who voice their little complaints in the *Daily Telegraph* strike a new note. They do not want much, but they think they have been overshadowed by their female compeers. It is quite true that thousands of men, over age or otherwise exempt from military duties, did work devotedly "at fatiguing and frequently nauseous tasks; and defrayed their own expenses, which were often heavy." They are wounded that they were not given a place in the Victory march, in common with female V.A.D. workers. They are disappointed that the B.R.C.S. has not given them medals for air-raid work. Lastly, they would like an official word in the rare event of being "mentioned," instead of having the news from a photographer. With every regard for these excellent male workers we cannot forbear a smile at the turn in the wheel of fortune. It seems but yesterday that the suffragettes were ringing the bell to emphasise the iniquitous manner in which the male part of the population managed to secure all the honours that were going. And now. . . .

In its endeavours to introduce the 48-hour working week to the general satisfaction of all, the Camberwell Board of Guardians sought the view of the nurses. A committee placed the requirements of the institution before the nurses, and discussed with them the desirability of the adoption of an eight-hour shift or longer weekly leave. The nurses unanimously decided in favour of the present duty hours and to have longer weekly leave.

